# Using protection motivation theory to explain intentions to vaccinate against tick-borne encephalitis

**DOI:** 10.1186/s12889-025-25470-6

**Published:** 2025-12-23

**Authors:** Sarah Eitze, Valeria Maslov, Leonie Otten

**Affiliations:** 1 Institute for Planetary Health Behaviour, University of Erfurt, Erfurt, 99089 Germany; 2 Bernhard Nocht Institute for Tropical Medicine, Hamburg, 20359 Germany

**Keywords:** PMT, Vaccine acceptance, Health behavior, Health psychology, Risk perceptions

## Abstract

**Background:**

In central Europe, tick-borne encephalitis (TBE) virus poses a growing health risk, amplified by climate-related changes in tick distribution. However, the TBE vaccination rate in Germany remains low. This study uses the Protection Motivation Theory (PMT) to investigate factors influencing TBE vaccination intention in Germany and the effect of tailored information on TBE risk areas.

**Methods:**

Participants (*N* = 309) aged between 18 and 61 living in Germany were included in an online survey with an additional experimental variation of tailored TBE risk messages. Three experimental groups were provided with (a) no tailored risk information, (b) semi-tailored information about risk areas on federal level or (c) full-tailored information on risk areas on county levels.

**Results:**

The PMT concepts showed that perceived susceptibility, severity, and response efficacy were positively related to the intention to be vaccinated against TBE. In exploratory analyses, perceived self-efficacy was a positive predictor of vaccination status. Permanent residence in risk areas was also associated with vaccination status, whereas planned or previous vacations in such areas were not. The effect of tailored information on vaccination intentions could not be confirmed.

**Conclusions:**

TBE vaccination intentions and uptake appear to be shaped by PMT factors and residence in risk areas. Public health interventions should therefore focus on communicating TBE risk, enhancing self-efficacy, and presenting clear evidence of vaccine efficacy to strengthen preventive measures against tick-borne diseases. Factors such as travel planning should also be considered in evidence-based health campaigns.

**Supplementary Information:**

The online version contains supplementary material available at 10.1186/s12889-025-25470-6.

## Using protection motivation theory to explain intentions to vaccinate against tick-borne encephalitis: evidence from an online survey

Vector-borne diseases account for more than 17% of all infectious diseases worldwide. They are caused by pathogens such as bacteria, viruses, or parasites transmitted by a vector. Vectors are living organisms capable of transmitting pathogens from person to person or from animal to person [1]. The impacts of climate change on the seasonal distribution of vector-borne diseases in Europe are increasing under scrutiny. Climatic factors, such as temperature, humidity, and precipitation patterns, influence the life cycle and reproduction rate of vectors, such as ticks, leading to a higher transmission risk through larger vector populations [2]. Long-term changes in seasons can also affect human activities and land use, further influencing the distribution and prevalence of vector-borne diseases in Europe [3].

In Germany, pathogens transmitted by ticks pose the most significant medical threat among the pathogens transmitted by blood-sucking vectors (such as mosquitoes, sandflies, lice, etc.) [4]. Climate can affect high-risk tick populations such as *Ixodes ricinus,* by influencing their mortality, reproduction, host populations, and habitat [5]. Warmer temperatures favor tick reproduction, prolong the tick season, and promote host-seeking behavior. At the same time, higher humidity increases tick survival rates. These changes lead to an expansion of tick populations and the diseases they transmit, such as tick-borne encephalitis (TBE). TBE is caused by three subtypes of the tick-borne encephalitis virus (TBEV)—European, Siberian, and Far-Eastern—which differ in their geographic distribution and clinical presentation. In Europe, almost all human TBE cases are caused by the European subtype, which will be the focus of this paper. TBE is a flu-like infection that typically occurs in two phases. The majority of individuals (approximately 70–95%) remain asymptomatic or do not progress to the second phase [6]. In symptomatic TBE infection, the illness typically follows a biphasic course: after an incubation period of 7–14 days, the first phase presents as a nonspecific febrile illness with symptoms like headache, body aches, and fever [7]. If the disease progresses to the second (neurological) phase of TBE, patients frequently develop meningitis, meningoencephalitis, meningomyelitis or meningoencephalomyelitis. Serious neurological signs—including peripheral or central paresis and cranial nerve palsies—can be observed [8].

Temperature increases due to climate change in the Northern United States and Europe have already led to a drastic expansion of Ixodes ticks and an associated increase in cases of Lyme disease and TBE [9, 10]. Moreover, rising temperatures in Europe have caused ticks to migrate to higher altitudes [3, 11].

The expansion of the distribution area of *Ixodes ricinus* ticks has also been documented in Germany [3]. Although ticks are widespread throughout Germany [4], there are high risk areas (HRA) of TBE infection, especially in Bavaria, Baden-Württemberg, southern Hesse, southeastern Thuringia, Saxony, and since 2022, southeastern Brandenburg [12]. The annual number of TBE cases also varies greatly. In 2024, a total of 686 cases of TBE were reported in Germany, representing a 44% increase compared to the previous year. In 559 cases, the infection was acquired within Germany, while the remaining cases were associated with travel abroad. The majority of patients (98%) were either unvaccinated or insufficiently vaccinated [13]. The Standing Committee on Vaccination (STIKO, [14]) recommends TBE vaccination for people living in or traveling to risk areas, as well as for certain occupational groups, starting from 1 year of age. The schedule is three doses for primary immunization, followed by boosters every 5 years (or every 3 years if aged 60 +). In Germany, TBE vaccination is generally provided by general practitioners (GPs) and, in some regions, also by pediatricians and local public health offices. In designated high-risk counties, the vaccination is free of charge for residents and for travelers to these areas; the costs are usually covered directly by the statutory health insurance without requiring reimbursement by the individual. Despite this, vaccination coverage in high-risk counties remains low, ranging from 6.5% to 26% among adults in 2022 (primary immunization plus possible booster vaccination; [13]). Barriers to vaccination often stem from low risk perception and fear of vaccine side effects [15]. Due to this complex situation, enhanced health communication is required. This should not only consider the knowledge and risk perception of the population regarding tick-borne diseases but also the motivation of people to adopt preventive behaviors.

Many studies on protection against tick-borne diseases focus on knowledge and risk perception of ticks and the associated diseases, as well as on how well people already protect themselves [15–17]. In the European context, three studies shed light on the psychological enablers and barriers of TBE vaccination. Hansen et al. [18] applied Protection Motivation Theory (PMT) across Denmark, Norway, and Sweden, the study identified that *perceived severity* of tick bites and Lyme borreliosis (LB) significantly predicted adoption of nonspecific protective behaviors. *Perceived vulnerability* (i.e., the belief in one’s likelihood of being bitten or contracting LB) had only a modest but significant influence. Notably, *response efficacy*—the belief that preventive measures are effective—was a strong predictor of protective behavior. Ates et al. [19] surveyed parents on TBE childhood vaccination, the study demonstrated that general pro-vaccine attitudes strongly correlated with willingness to vaccinate against TBE. *Perceived response efficacy* and *perceived safety* of the TBE vaccine were important enabling factors, while barriers included cost concerns and exposure to misinformation. At last, Riccò et al. [16] revealed in a questionnaire-based survey among farmers in the Autonomous Province of Trento, Italy, that general knowledge about tick-borne diseases was fairly low, but 73.6% perceived TBE as a frequent threat nevertheless and 61.3% perceived TBE as severe. Only 43.3% knew about the TBE vaccine, and just 24.5% had been vaccinated.

To develop effective preventive measures, it is important to structurally understand what motivates people to adopt such behaviors as the TBE vaccine. In this regard, the Protection Motivation Theory (PMT, [20]) is useful, because it includes previously found factors such as perceived severity (of TBE) and response efficacy (of the vaccine). The PMT explains how motivation shapes protective behaviors in response to potential threats. The theory has been widely applied in the vaccination context, for example, to generalized vaccination demands [21], to COVID-19 vaccinations [22, 23], or with regard to other specific vaccinations such as influenza [24], but evidence for TBE is limited.

**Fig. 1:**
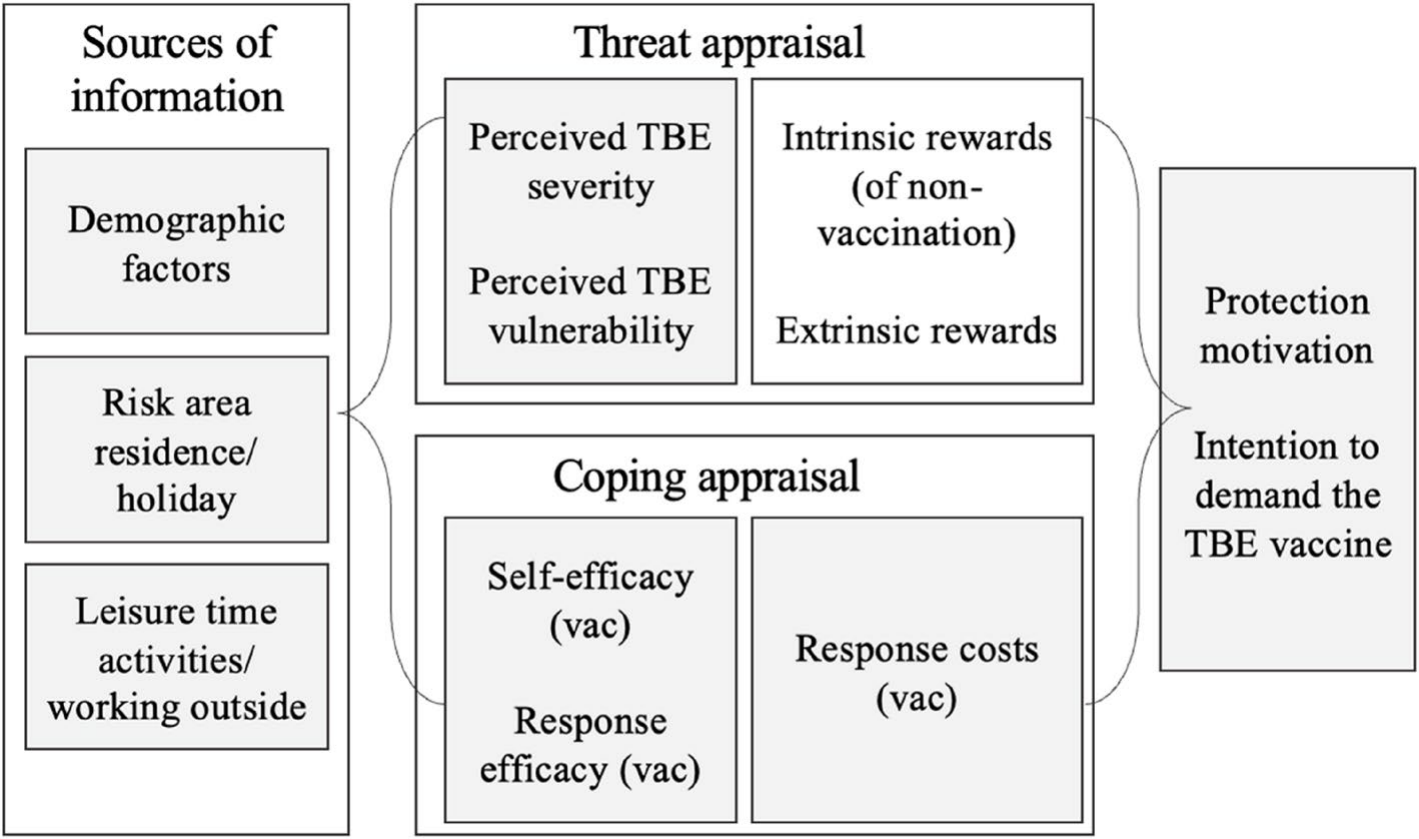
Theoretical overview of the PMT constructs explaining intention to vaccinate against tick-born encephalitis (TBE) Note. Factors highlighted were measured in this study

We aim to identify the factors influencing the intention to vaccinate against TBE, with a particular focus on examining the impact of the PMT dimensions (perceived severity, perceived susceptibility, response efficacy, and self-efficacy) on vaccination intentions. Figure 1 shows the PMT adaptation to TBE vaccination and highlights, which of the dimensions we measured in our study.

Additionally, we will investigate the effect of using tailored health information about high-risk areas on vaccination intention. Risk perceptions generally encompass both cognitive and affective components [25, 26]. The cognitive component includes an individual’s assessment of the likelihood of occurrence and the perceived severity of a negative event. Providing information about high-risk areas for tick-borne encephalitis (TBE) is expected to increase the perceived probability of infection and, consequently, strengthen the intention to vaccinate, as predicted by PMT [20]. The findings of this study could help identify influencing factors for vaccination intentions and strategically apply them in communication about TBE vaccination to increase vaccination rates in vulnerable population groups.

## Hypotheses

All hypotheses and analyses were pre-registered and are available in the online repository of the Open Science Framework (OSF):

- (https://osf.io/xg2r5/).

### Threat appraisal hypothesis

In the context of TBE vaccination, the core concepts of threat appraisal within the PMT include the perceived severity of TBE and the belief that one is susceptible to infection. It is assumed that perceived severity and susceptibility are positively associated with the intention to vaccinate against a disease [27, 28].

*H1a: The higher the perceived severity of TBE, the higher the intention to vaccinate against TBE.*

*H1b: The higher the perceived susceptibility to TBE, the higher the intention to vaccinate against TBE.*

### Coping appraisal hypothesis

Another central component of PMT is coping appraisal, which consists of self-efficacy and response efficacy [20]. In the context of TBE vaccination, self-efficacy refers to an individual’s belief in their ability to successfully implement the recommended protective measure, the TBE vaccination. Response efficacy refers to the belief that TBE vaccination is effective in protecting against TBE infection. Studies show that perceived self-efficacy and response efficacy are positively associated with the intention to vaccinate against diseases (like COVID-19: [29]; or influenza: [23]).

*H2a: The higher the perceived self-efficacy in obtaining the TBE vaccine, the higher the intention to vaccinate against TBE.*

*H2b: The higher the perceived efficacy of the TBE vaccine (response efficacy), the higher the intention to vaccinate against TBE.*

### Response costs hypothesis

Maladaptive response rewards and response costs are both categorized under the broader term of response costs [30]. Response costs in the context of TBE vaccination refer to the associated expenses (time, effort, etc.). Maladaptive response rewards denote the perceived benefits of abstaining from TBE vaccination, such as avoiding potential costs. Research indicates that individuals are more inclined to vaccinate when they perceive fewer rewards for not doing so [28, 31]. Similarly, Griffin et al. [27] found that lower maladaptive response rewards and response costs were linked to a stronger intention to receive the COVID-19 vaccine.

*H3a: The higher the perceived maladaptive response rewards for not vaccinating against TBE, the lower the intention to vaccinate.*

*H3b: The higher the perceived response costs of TBE vaccination, the lower the intention to vaccinate.*

### Message tailoring hypothesis

Studies, including meta-analyses, have shown that tailored messages are more effective than generic ones, particularly regarding health-related behaviors. This is because such messages are perceived as more relevant to the individual and are therefore more likely to be noticed and followed [32–34]. Additionally, an effect of tailored communication has been observed in the context of vaccinations [35, 36].

*RQ2: What influence does tailoring TBE information have on the intention to vaccinate against TBE?*

*H4: Providing tailored information about TBE risk areas leads to a higher willingness to vaccinate against TBE compared to providing general information about TBE risk areas or no specific information about TBE risk areas (control group).*

## Methods

**Fig. 2:**
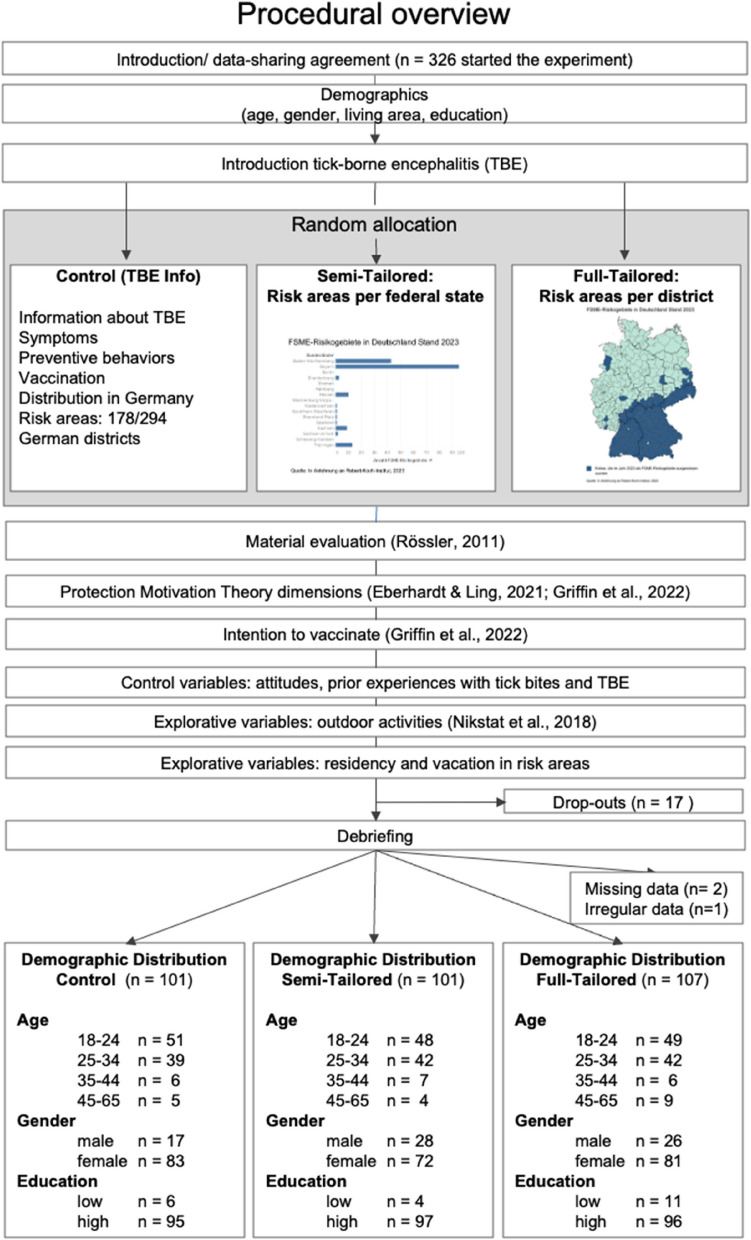
Procedural overview for the online experimental survey Note. The single boxes in the flow chart represent pages of the online experiment. When validated scales such as the outdoor activities scale [37] are used, references are shown as well

The study had a single-factor between-subject design with three levels: the factor TBE information was varied as (1) control condition, (2) semi-tailored information, (3) and full-tailored information. Figure 2 shows a measurement overview, procedure, and demographic information for the experimental subgroups. Further participants’ characteristics are displayed in Supplementary Table 1. Full measurements, the consent form, and questionnaires are openly available on OSF (https://osf.io/xg2r5/).

### Sample

Between 29.10.—20.11. 2023, *N* = 306 participants answered an online questionnaire (EFS survey by Questback). Participants were recruited through social media, SurveyCircle, and the Erfurt Laboratory for Empirical Research at the University of Erfurt. We excluded participants who (1) did not reside in Germany, and (2) were younger than 18 years. Participants’ mean age was *M* = 26.8 (*SD* = 8.40, [18–61]); 76.5% were female. The majority (93.5%) had at least 10 years of schooling with qualification for higher education; and 86.9% lived in urban areas. Participants were randomly assigned to one of the three study conditions; with *n* = 100 (32.7%) in the control condition, *n* = 100 (32.7%) received generic information, and *n* = 106 (34.6%) the tailored information.

The questionnaire was compiled from the literature for this survey and can be found in Supplement 2. Since the official recommendation made by the German STIKO for TBE vaccination is to administer three doses for primary immunization and then a booster dose every 10 years [14], the vaccination status is correspondingly displayed in six categories. Participants could indicate whether they had received one (*n* = 5), two (*n* = 6), or three doses (full primary immunization, *n* = 51) of TBE-vaccination, whether they had received a booster (*n* = 62), whether they had not yet been vaccinated against TBE (*n* = 147), or whether they were unable or unwilling to provide this information (*n* = 38). For further analyses, this variable was recoded in vaccinated (*n* = 123) and unvaccinated (*n* = 147), participants with unclear status were excluded from the analyses.

### Measurements

The questionnaire was developed by compiling scales for the PMT constructs from the literature. When more than one option was available for a given construct, the scale with the higher reported validity was selected, where such information was provided. All scales were adapted to the context of TBE and translated into German. The translation was carried out by native German speakers with the assistance of DeepL-AI. The questionnaire was pretested for comprehensibility and technical implementation by five students of the master program in Health Communication, and suggested modifications were incorporated. Internal consistency was assessed using Cronbach’s alpha, with values above.70 generally considered acceptable for research purposes [38].

The dependent variable, the *intention to vaccinate* against TBE was assessed with three translated and modified items from Eberhardt & Ling [31] and Griffin et al. [27]. On a 7-point Likert scale, participants indicated their agreement to statements like “I am willing to get vaccinated against TBE”. Cronbach’s Alpha was α =.91.

*Severity of TBE* comprised three translated and modified items from Eberhardt & Ling [31] and Griffin et al. [27], e.g. “The negative effects of TBE are very serious”. We calculated a mean score (*1* = definitely not agree to *7* = definitely agree; Cronbach’s α =.72).

Participants rated their *Susceptibility for TBE* on a 7-point Likert scale ranging from 1 = “definitely do not agree” to 7 = “definitely agree.” The scale comprised three items, such as “If I am not vaccinated against TBE, I am at risk of getting infected with TBE.” Cronbach’s alpha was α =.51, indicating low internal consistency. Therefore, an additional reliability assessment was performed using McDonald’s Omega, which provides a more accurate estimate of scale reliability, particularly for small scales or when assumptions of tau-equivalence are violated. Based on the Omega analysis, the most reliable item was selected for further analyses.

*Response Efficacy* and *Self Efficacy* were each measured with three translated and modified items from Eberhardt & Ling [31] and Griffin et al. [27] and focused on the vaccination against TBE. On a 7-point Likert scale from *1* = definitely not agree to *7* = definitely agree, participants rated statements like “It would be very easy for me to get vaccinated against TBE”. For self efficacy, we used the mean score (Cronbach’s α =.86). For response efficacy (with Cronbach’s α =.64), for further analyses, we chose the item “A TBE vaccination would protect me from getting TBE” based on Omega results.

Three items like “If I don’t get vaccinated against TBE, it has benefits for me” [27]; translated and adapted) assessed *Maladaptive Response rewards* on a 7-point Likert scale from *1* = definitely not agree to *7* = definitely agree. Cronbach’s Alpha was α =.42. Based on Omega, we decided to use the item “If I’m not vaccinated against TBE, I don’t have to spend time and effort on the vaccination” in further analyses.

Participants indicated *Perceived response costs* with three translated and adapted items from Xiao et al. [23], using a 7-point Likert scale (*1* = low costs, *7* = high costs). Cronbach’s Alpha was α =.52. Omega analysis suggested the item “The TBE vaccination would be inconvenient for me” as the most appropriate for further analyses.

The evaluation of the informational material is covered by Rössler’s [39] scale. It comprises 6 items. Using a 5-point semantic differential scale, participants indicate whether the material is perceived as interesting/uninteresting, understandable/unintelligible, personally relevant/irrelevant, convincing/not convincing, serious/not serious, and well/poorly made. The item of personal relevance is utilized as a manipulation check, as responses should differ between control and tailoring groups.

### Procedure

Prior to the survey, all participants gave informed consent, and we informed the participants that they can end the questionnaire at any time. At the beginning of the survey, all participants received some general information about TBE including: a definition of TBE, description of symptoms and the course of the disease, ways to protect against TBE including TBE vaccination. The information was based on existing health information from the Federal Centre for Health Education (BZgA) [40], the BZgA and the Federal Association of Physicians in the Public Health Service, the Institute for Quality and Efficiency in Health Care (IQWiG) [41], and the Robert Koch-Institute (RKI) [12].

The second part of the information material explained the spread of TBE in Germany and included three groups for the tailoring experiment. In the control condition, participants read a description of the current number of risk areas and in which regions they are located.

In the semi-tailored condition, the number of risk areas was presented in a bar chart, indicating the total number of risk areas per federal state as of 2023. In contrast to the full-tailored information, it did not specify smaller risk areas. The full-tailored condition provided a choropleth map that highlighted the TBE risk areas in Germany as of 2023 colored in blue. Information materials were based on annually published data from the Robert Koch-Institute (years shown: 2005, 2014, 2023) [14]) and visualized with the software Tableau Public.

### Statistical analyses

TBE vaccination, as recommended by the RKI, needs to be repeated with one dose every 10 years. Therefore, we calculated a regression analysis including all PMT dimensions and socio-demographic variables for the complete sample, but also for the subsamples of vaccinated and unvaccinated participants. To test the influence of tailoring TBE information on the intention to vaccinate against TBE (H4), a one-way analysis of variance (ANOVA) was conducted with TBE vaccination intention as the dependent variable and the type of information provision about TBE high risk areas (full-tailored, semi-tailored, and control condition) as the independent variable.

## Results

All analyses were performed using R Studio (R Version 4.3.2). Raw data and analysis scripts are openly available on OSF (https://osf.io/xg2r5/). Before conducting the multiple linear regression analyses, the necessary statistical assumptions were examined. Visual inspection suggested that the relationships between independent and dependent variables were linear. Homoscedasticity of residuals was confirmed using the Breusch–Pagan test (*p* >.284), indicating that the variance of residuals was constant and standard errors were not biased. Cook’s distances were all below the conventional cut-off of 1 (M = 0.014), suggesting that no influential outliers distorted the model. The Durbin–Watson statistic (1.94) indicated no autocorrelation in the residuals. Multicollinearity among predictors was excluded as all VIF values were below 10, ensuring stable coefficient estimates. Residuals were approximately normally distributed according to the Shapiro–Wilk test (*p* >.159), supporting the validity of the significance tests. In addition, as data were collected from individual respondents, independence of observations could be assumed.

To further evaluate the adequacy of the sample size and the statistical power of the regression, a post-hoc power analysis was conducted using G*Power. For an observed effect size of f^2^ = 0.71, with *N* = 100 participants and six predictors, the computed power was 99%, indicating high statistical power for detecting the reported effects. Taken together, these results indicate that no substantial violations of the regression assumptions were present, supporting the validity and reliability of the reported regression estimates, standard errors, and significance tests.

### PMT dimensions and the intention to vaccinate

In hypotheses H1-H3, we assumed that the dimensions of the PMT would be associated with the intention to vaccinate against TBE. Table 1 shows the results for a multiple linear regression including all dimensions and basic socio-demographic variables.

**Table 1:** Multiple linear regression predicting the intention to vaccinate using PMT Factors and socio-demographic control variables

Variables	**Unvaccinated** ***n* = 145**			**Vaccinated** ***n* = 161**			**Complete Sample** ***n* = 306**		
	std. Beta	standardized CI	*p*	std. Beta	standardized CI	*p*	std. Beta	standardized CI	*p*
Age	−0.14	−0.31–0.03	0.104	−0.09	−0.24–0.06	0.215	−0.16	−0.26 – −0.06	**0.003**
Gender (female vs. male)	−0.15	−0.52–0.21	0.398	0.01	−0.36–0.38	0.960	−0.04	−0.26–0.19	0.744
Education (high vs. low)	−0.11	−0.81–0.59	0.761	−0.34	−0.88–0.20	0.213	0.03	−0.35–0.42	0.869
Susceptibility (TBE)	0.20	0.02–0.37	**0.028**	0.12	−0.04–0.27	0.140	0.17	0.06–0.28	**0.002**
Severity (TBE)	0.17	0.01–0.34	**0.036**	0.19	0.04–0.33	**0.015**	0.17	0.07–0.28	**0.001**
Self-efficacy (Vac)	−0.05	−0.23–0.12	0.556	0.42	0.26–0.59	**< 0.001**	0.15	0.04–0.27	**0.007**
Response efficacy (Vac)	0.19	0.03–0.36	**0.022**	0.22	0.07–0.36	**0.004**	0.23	0.13–0.34	**< 0.001**
Response costs (Vac)	−0.13	−0.31–0.05	0.153	−0.15	−0.30–0.01	0.059	−0.17	−0.28 – −0.06	**0.002**
Non-response benefits (Vac)	0.00	−0.17–0.17	0.965	0.01	−0.14–0.16	0.852	−0.02	−0.12–0.09	0.767
Observations	145			123			306		
R^2^/R^2^ adjusted	0.178/0.123			0.505/0.466			0.338/0.318		

Susceptibility and Severity were asked for the disease tick-borne encephalitis (TBE), the four other dimensions were asked for the vaccination (Vac). The unvaccinated sample reported to have never received one vaccine against TBE. In the complete sample, we included all participants, because TBE vaccination is recommended to be repeated every 10 years

The results of the linear regression for the complete sample suggested that perceived susceptibility to TBE and the perception of the severity of an TBE infection, as well as self-efficacy, response efficacy, and response costs were associated with increased intention to vaccinate. For previously vaccinated participants, only perceived severity, self-efficacy, and response efficacy were significantly associated with vaccination intentions. In the unvaccinated participants, perceived susceptibility and perceived severity, as well as response efficacy were significant predictors for vaccination intentions. In conclusion, this analysis partially supported both the threat appraisal hypothesis (H1) and coping appraisal hypothesis (H2), while the response cost hypothesis (H3) was confirmed only in one out of six predictor tests.

### Tailoring health information

**Fig. 3:**
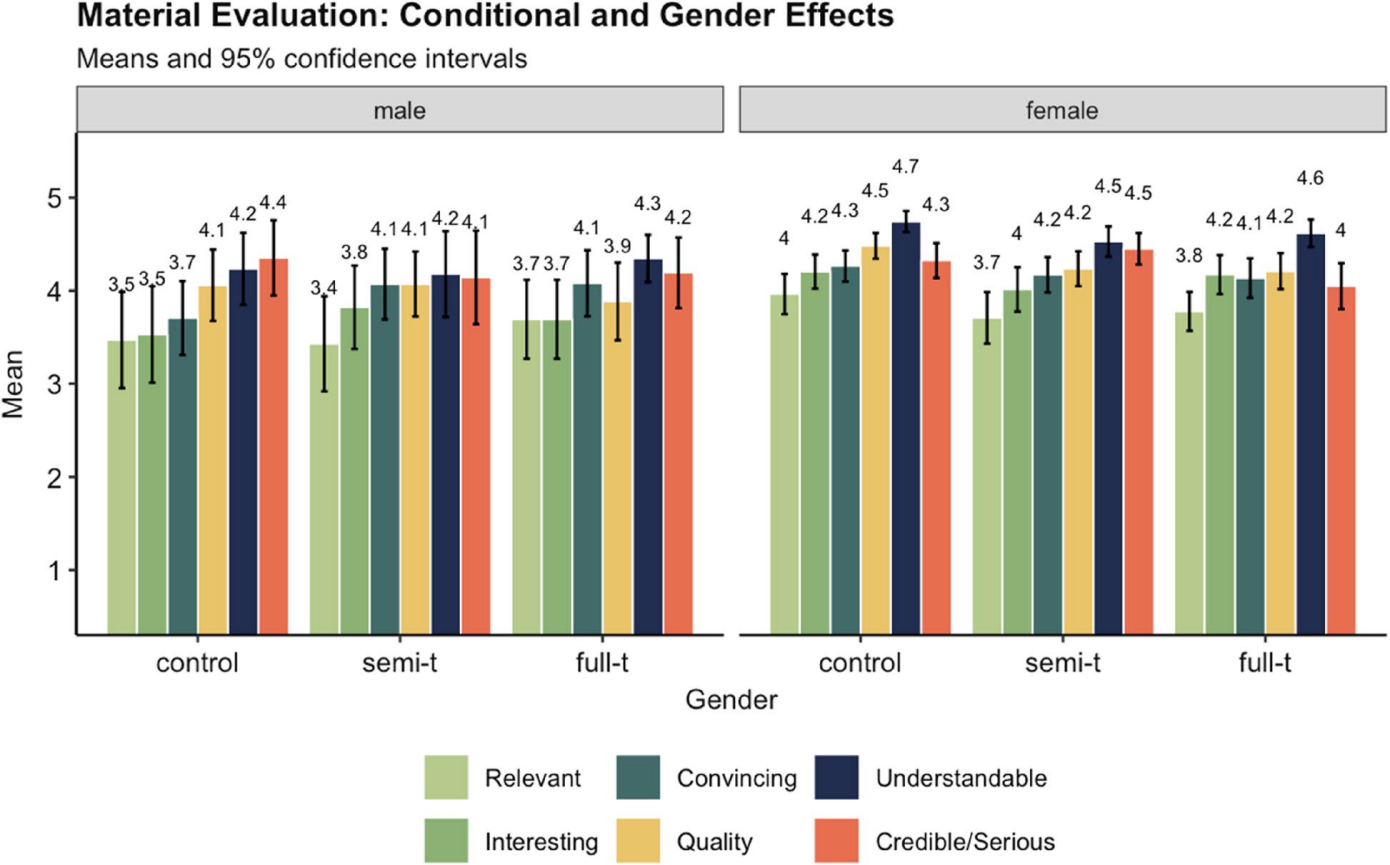
Evaluation of the information in the experimental conditions, differences between male and female participants Note. The semantic differentials were presented on 5-point scales. The question of relevance (light green) served as a manipulation check. In general, female participants rated the information more positive compared to male participants. There was no interaction with experimental condition

To assess whether the experimental manipulation was successful, a manipulation check for the item “I perceive the material as personally relevant” was conducted. After checking the assumptions, a one-way ANOVA was performed to test differences in the perceived personal relevance of the TBE information among the experimental groups. The results revealed no significant difference between the experimental groups, *F*(2, 303) = 0.974, *p* =.379, η2 =.001. This outcome suggests that the manipulation was not successful in terms of relevance. Interestingly, including gender in the analyses of the evaluation revealed that female participants perceived the information material as more interesting and more understandable in comparison to men. A complete representation of all evaluation items between conditions and gender can be seen in Fig. 3.

For the ANOVA regarding vaccination intentions and high-risk information, the assumptions were checked initially. According to the Levene test, equality of variances could be assumed (*p* =.073). However, according to the Shapiro–Wilk test, normal distribution could not be assumed (*p* <.001). Nonetheless, an ANOVA was conducted as the one-way ANOVA is relatively robust to violations of the normal distribution assumption, especially with equal group sizes [42–44].

**Fig. 4:**
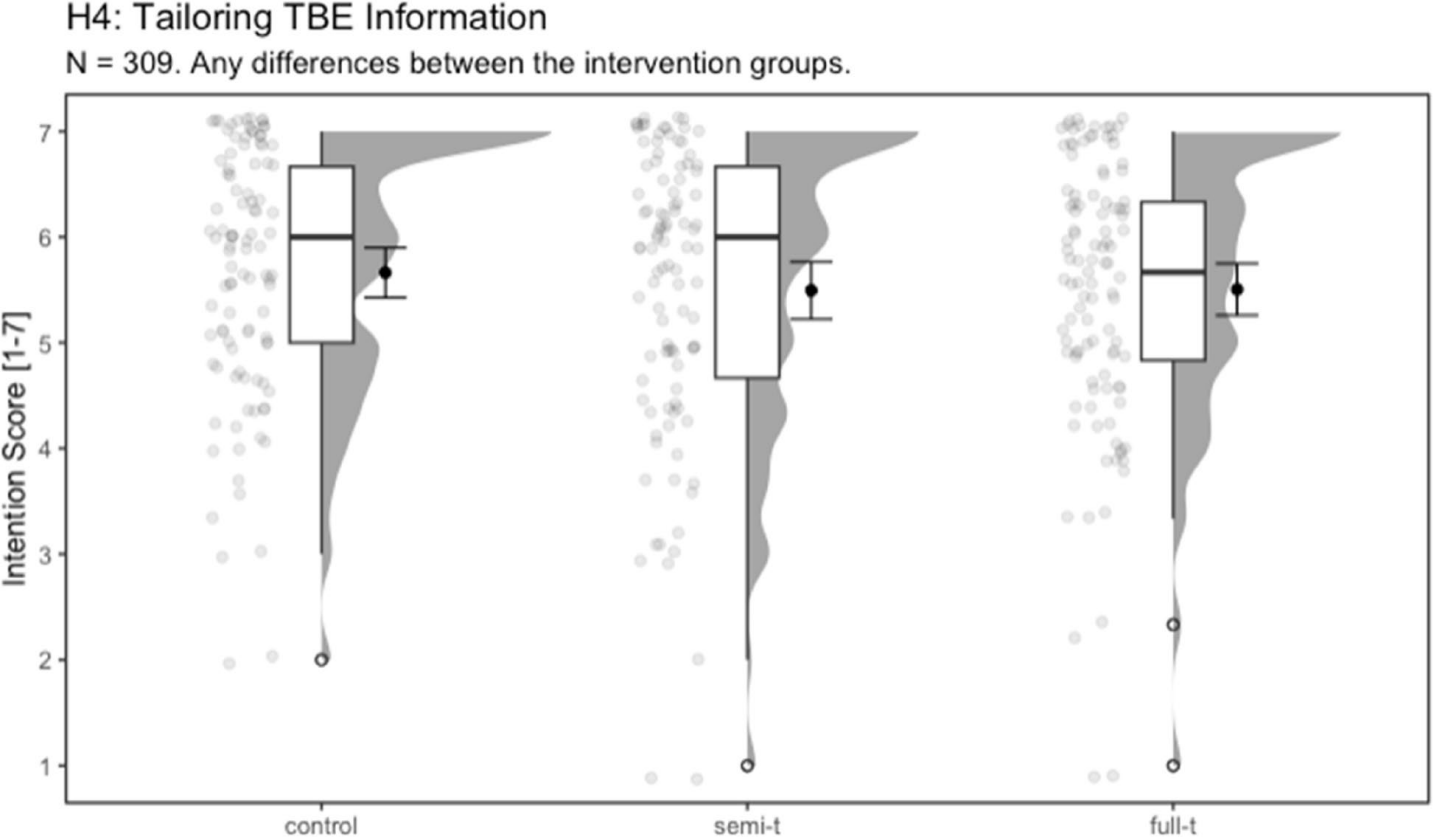
Experimental influence on the intention to vaccinate against TBE Note. Intention to vaccinate against TBE after the online experiment in the control condition, the semi-tailored condition using federal states (semi-t), and the full-tailored condition displaying information on the district level (full-t). There were no significant differences between the experimental conditions

The ANOVA revealed no significant difference between the control condition (*n* = 101, *M* = 5.66, *SD* = 1.20), the semi-tailored condition (*n* = 101, *M* = 5.50, *SD* = 1.38), and the full-tailored condition (*n* = 107, *M* = 5.51, *SD* = 1.28). Thus, the provision of tailored information about TBE risk areas had no positive impact on TBE vaccination intention, leading to the rejection of H4, *F*(2, 300) = 0.362, *p* =.696, η2 =.002. Figure 4 shows distributions of the dependent variable in the experimental conditions.

### Explorative analyses: PMT dimensions and the vaccination status

In an exploratory logistic regression, the relationship between the PMT dimensions and self-reported vaccination status was examined. *N* = 41 participants could not indicate their vaccination status (they chose the “don’t know” option as an answer) and are excluded from this analysis. In the sample available for logistic regression (*N* = 268), the proportion of individuals vaccinated against TBE was *n* = 123 (45.89%). Overall, the variance explained by the model with PMT dimensions and sociodemographic variables (age, gender, and education) was R^2 = 0.184. With higher self-efficacy, the likelihood of vaccination significantly increases (OR = 1.87 [1.41–2.54]). Apart from self-efficacy, a significant association with participants’ age was observed. The younger the participants, the greater the likelihood that they also received a TBE vaccination (OR = 0.95 [0.91–0.98]). Trends without significant associations were observed for gender (female) (OR = 1.66 = [0.86–3.27]) and severity (OR = 1.15, [0.92–1.45]). Susceptibility, response efficacy, response costs, or education also show no significant association with the participants’ vaccination status. In a second analysis, including information about the high-risk areas, only permanent residence in a high-risk area was associated with the vaccination status of the participants, whereas future or past holidays in a high-risk area were not associated with the vaccination status. The results are presented in Table 2.

**Table 2:** Logistic regression predicting vaccination status using PMT Factors, sociodemographic variables and risk area relevance

Predictors	**Vaccination Status:** **PMT Model**			**Vaccination Status:** **PMT + Risk areas**		
	Odds Ratios	CI	*p*	Odds Ratios	CI	*p*
Age	0.95	0.91–0.98	**0.003**	0.94	0.89–0.98	**0.008**
Gender (female vs. male)	1.66	0.86–3.27	0.133	1.91	0.88–4.28	0.109
Education (high vs. low)	0.58	0.19–1.74	0.323	0.72	0.21–2.48	0.599
Self-efficacy (Vac)	1.87	1.41–2.54	**< 0.001**	1.77	1.24–2.60	**0.002**
Severity (TBE)	1.15	0.92–1.45	0.227	1.20	0.90–1.62	0.215
Susceptibility (TBE)	1.07	0.88–1.31	0.503	1.04	0.80–1.34	0.788
Non-response benefits (Vac)	0.97	0.85–1.12	0.699	1.04	0.86–1.24	0.702
Response efficacy (Vac)	1.02	0.81–1.28	0.863	1.10	0.81–1.47	0.545
Response costs (Vac)	0.94	0.79–1.11	0.444	0.86	0.69–1.07	0.180
Past vacation in HRA (no vs. yes)				0.85	0.34–2.16	0.727
Future vacation in HRA (no vs. yes)				0.59	0.24–1.44	0.246
Living in HRA (no vs. yes)				0.19	0.09–0.37	**< 0.001**
Observations	268			212		
R^2^ Tjur	0.184			0.310		

Vaccination status was recoded as (1) started or completed TBE vaccination scheme vs. (0) did not receive any TBE vaccination dose. Susceptibility and Severity were asked for the disease tick-borne encephalitis (TBE), the four other dimensions were asked for the vaccination (Vac)

## Discussion

The aim of the present study was to investigate the influence of PMT constructs on individuals’ intention to vaccinate against TBE. The study further examined the impact of tailored information about TBE risk areas in Germany, while also considering the influence of demographic data, vaccination attitudes, exposure to risk areas, and previous TBE vaccination behavior. In terms of vaccination intention, the pre-registered analysis revealed that higher perceived severity and susceptibility to TBE, as well as greater response efficacy, were associated with a stronger intention to vaccinate. Exploratory analyses comparing vaccinated and unvaccinated participants showed that self-efficacy and residing in high-risk areas were significantly related to vaccination status. Additionally, younger age was associated with a higher likelihood of having received a TBE vaccination.

Based on our pre-registered hypotheses and the explorative analysis, we find that the dimensions of the Protection Motivation Theory outlined above are instrumental in explaining TBE vaccination intentions and behavior within a convenience sample in Germany. However, it is noteworthy that non-response benefits did not emerge as a significant predictor in any of the analyses. Additionally, while response costs exhibited a significant association only when considering the entire sample in the analysis of vaccination intention, its impact needs further investigation. The present study also examined the influence of depicting increasing TBE risk areas on vaccination intention. The results indicated no change in perceived susceptibility and TBE vaccination intention when an increase in risk areas in Germany was depicted. Overall, the PMT explained 42% of the variance in TBE vaccination intention in the present study. The coefficient of determination was therefore somewhat lower compared to previous research on vaccination intentions against COVID-19 and influenza, where PMT factors alone accounted for 62% [45] and 76% [28] of the variance in vaccination intention, respectively. This finding suggests that, although PMT also makes a substantial contribution to explaining TBE vaccination intention, other factors may play a more prominent role, or the dynamics between different determinants may differ for TBE vaccination compared to other infections such as COVID-19 and influenza. Further research would be valuable to better understand the specific differences and similarities in the role of PMT across various vaccination decisions.

From the exploratory analyses of self-reported vaccination status, we learn that both self-efficacy and a younger age are associated with a higher likelihood of receiving the vaccination. The age effect contradicts other research findings, so it’s important to discuss the results. One possible cause of this effect is the interplay of two factors. Firstly, the sample in this study is significantly younger than the general population. Consequently, the effect of the vaccines’ recommendation explicitly for people beyond the age of 60 cannot be reflected here. Secondly, the results may suggest that immunization for children has been gradually increasing over the last decades due to interventions and increased focus by physicians. Future studies should further examine this effect in younger cohorts to additionally confirm these promising findings.

Even though residing in high-risk areas is associated with vaccination status, planning or spending a holiday in a high-risk area was not. Given that increased exposure to nature during vacations often implies a heightened risk of TBE, this finding serves as a starting point for enhancing vaccination rates where they are most likely to prevent diseases: among young individuals who engage in outdoor activities. This vaccination initiative could be combined with an intervention providing general information on tick protection, aiming to reduce tick bites overall and prevent further associated diseases such as Lyme disease.

The non-significant results of the experimental manipulation do not necessarily mean that depicting the high-risk areas has no influence at all. It is possible that the chosen method of information presentation was not robust enough to induce a change in vaccination intention and perceived susceptibility.

### Strengths and limitations

The present study exhibits several limitations that may affect the conclusions drawn from the results, which are discussed below. Firstly, recruitment for the online survey was conducted via social media, the Erfurt Laboratory at the University of Erfurt, and the online platform SurveyCircle, resulting in a sample bias towards younger, more educated, and urban individuals, thus compromising the generalizability of the findings. Additionally, the recruitment methods may have introduced self-selection bias, with individuals holding very positive attitudes towards vaccinations being more inclined to participate in the study. On the other hand, the relations that are found even in this sample might be even of higher effect size in a general population sample displaying more variance in the intention and even vaccine hesitancy. A more balanced gender ratio would be advantageous. While a study by Grgič-Vitek & Klavs [46] on factors influencing TBE vaccination uptake in Slovenia found no gender-specific differences among those receiving paid TBE vaccinations, Hansen et al. [18] observed gender disparities in protection motivation. Therefore, it would be beneficial to investigate whether the present findings hold true in a sample with a more even gender distribution.

Another significant aspect to consider in interpreting the results is the influence of experimental manipulation. The manipulation check, which assessed the perceived personal relevance of the TBE information across experimental groups, revealed no significant differences, suggesting that the experimental manipulation was unsuccessful. The lack of effect of tailoring on vaccination intention could have various causes. One explanation would be that the provided information was not sufficiently personalized or personally relevant enough to induce a significant change in vaccination intention [18, 47]. Another explanation would be that people with no planned or permanent residences in risk areas consider the information as irrelevant or their personal vaccination choice. A more detailed analysis of the specific elements of tailored information and their potential influence could provide further insights in future studies.

With regard to divergent and content validity, we relied on evidence from previous studies that had developed and validated these scales in related contexts. Furthermore, the somewhat low internal consistency and consequently limited reliability of some scales related to PMT factors should be acknowledged. This particularly pertains to scales assessing perceived susceptibility, maladaptive response rewards, and response costs. In contrast, the underlying studies from which these scales were adopted [23, 27, 31] mostly rated internal consistency as at least acceptable. This raises questions about the low reliability observed in the present study. It is possible that the translation of items into German or their adaptation to the specific topic of TBE was not optimal. Therefore, future research should consider validating the scales with methodological studies to improve their applicability and reliability for the German population and the research context of TBE.

### Implications for future research and practice

Despite its limitations, the present study is among the first to examine the influence of PMT and tailoring information on TBE vaccination intention. Therefore, it provides important insights into potential directions for future research and highlights considerations for the development of measures and campaigns to promote TBE vaccination. Based on the theoretical framework of PMT, the present study identified factors associated with the intention to vaccinate against TBE. As a next step, future studies could initially investigate whether manipulating PMT constructs and the associated changes in these constructs are causally linked to changes in TBE vaccination intention and whether such changes can successfully lead to an increase in vaccination rates. Future studies might also benefit from improving the risk-area analysis by asking for county-level information. Researchers could also investigate whether there is cross-vaccine hesitancy in certain high-risk areas that would indicate the need for targeted local interventions including more general information about vaccination and risk perceptions. Evidence-based campaigns then need to be evaluated. If campaigns aimed at increasing vaccination rates include measures to enhance perceived susceptibility, self-efficacy among vaccine recipients, perception of the severity of TBE, and perceived efficacy of the TBE vaccine, their impact on vaccination uptake should be tested.

In addition to TBE, Lyme borreliosis is considered the most common vector-borne disease in temperate climates of the northern hemisphere (i.e., large parts of North America, Europe, Asia, as well as parts of Australia and South America). There are promising vaccine candidates currently under research, but not yet available to the public [48]. Future research could therefore investigate whether PMT can be applied not only to vaccination intention but also to the adoption of nonspecific preventive measures against tick bites in Germany, similar to what Hansen et al. [18] have already explored in Denmark, Norway, and Sweden. In their study, Hansen et al. found that intentions to adopt nonspecific preventive measures (e.g., protective clothing, tick checks) were generally higher than intentions to get vaccinated, suggesting that public health messaging could benefit from addressing both types of preventive behavior.

As more and more risk groups emerge by climate change, the question of travel vaccination might be of less interest than identifying the decisional influences of people living in risk areas and the interaction with leisure time activities such as hiking or camping that further increase the risk of tick-encounters. This might further inform evidence-based campaign development and offer ways to distribute information about TBE, for example within hiking groups, to use mountain guides that lead hikes as distributors, or include information about TBE in informational leaflets of campsites or nature reservoirs.

In our study, A study by Caputo et al. (2019) on the implementation of preventive measures against tick-borne diseases revealed deficiencies in knowledge about which pathogens are covered by the TBE vaccine. Additionally, there was a lack of implementation of nonspecific prevention measures. Climate change will exacerbate the TBE problem and tick bites in general. Therefore, it is of utmost relevance to public health that knowledge, risk perception, and vaccine willingness increase in the long term. These knowledge gaps highlight the need for clear and targeted communication strategies. Practical implications include improving vaccination documentation and record-keeping (e.g., digital vaccination certificates), integrating TBE-related information into routine consultations with general practitioners, and providing accessible educational materials on ticks, TBE infection, and vaccination through public health campaigns [49]. Strengthening health literacy in this area may help individuals make informed decisions and increase adherence to protective behaviors [50].

## Conclusion

In conclusion, the present study investigated the influence of Protection Motivation Theory (PMT) constructs on individuals’ intention to vaccinate against Tick-Borne Encephalitis (TBE) in a German convenience sample, while also considering the impact of tailored information about TBE risk areas and demographic factors. The findings are specific to a relatively young, female, highly educated, and largely urban sample, and may not fully reflect the perspectives of older adults, rural residents, or other segments of the general population. The findings suggest that higher perceived severity and susceptibility to TBE, along with greater response efficacy, are associated with a stronger intention to vaccinate. Additionally, self-efficacy and residing in high-risk areas are significant predictors of vaccination status. In contrast, planning or having spent a holiday in high-risk areas was no significant predictor of vaccination status, which clearly shows that TBE vaccination and travel preparation needs to be linked more closely by public health interventions. Future research could delve deeper into causal links between PMT constructs and vaccination intention, replicate the findings in additional target groups such as older adults or rural populations, as well as explore the applicability of PMT to non-specific preventive measures against tick bites. Given the increasing relevance of tick-borne diseases due to climate change, efforts to improve knowledge, risk perception, and vaccine willingness are crucial for public health in the long term.

## Supplementary Information

Supplementary Material 1.

Supplementary Material 2.

## Data Availability

Raw data and analysis scripts are openly available on OSF (https://osf.io/xg2r5/).
