# Supplementary material for: Using protection motivation theory to explain intentions to vaccinate against tick-borne encephalitis
Source: BMC Public Health. 2025 Dec 23;25:4345. doi: 10.1186/s12889-025-25470-6 (PMC12751593; doi:10.1186/s12889-025-25470-6)
Supplement: Supplementary file 1 — Supplementary Material 1. [file 12889_2025_25470_MOESM1_ESM.docx]

**SUPPLEMENT**

**Table 1. Descriptive Statistics**

| ***Variable*** | | ***N (%)*** | ***M (SD)*** |
| --- | --- | --- | --- |
| **Age** | |  | 26.78 (8.40) |
| **Gender** | |  |  |
|  | male | 70 (22.9%) |  |
|  | female | 234 (76.5%) |  |
|  | divers | 2 (0.7%) |  |
| **Education** | |  |  |
|  | up to 9 years | 3 (1.0%) |  |
|  | 10 years or more | 17 (5.6%) |  |
|  | >10 years with university qualification | 286 (93.5%) |  |
| **Living** | |  |  |
|  | Urban | 40 (13.1%) |  |
|  | Rural | 266 (86.9%) |  |
| **Self-reported vaccination status** | |  |  |
|  | Vaccinated | 124 (40.5%) |  |
|  | Unvaccinated | 145 (47.5%) |  |
|  | Unclear (don’t know) | 37 (12.1%) |  |
| **Vaccination Attitudes** | |  | 5.56 (1.29) |
| **Tick bite experiences** | |  |  |
|  | Yes | 190 (62.1%) |  |
|  | No | 103 (33.7%) |  |
|  | Unclear (don’t know) | 13 (4.2%) |  |
| **TBE-disease** | |  |  |
|  | Yes | 14 (4.6%) |  |
|  | No | 281 (91.8%) |  |
|  | Unclear (don’t know) | 11 (3.6%) |  |
| **Living in a high risk area (HRA)** | |  |  |
|  | Yes | 112 (36.6%) |  |
|  | No | 189 (61.8%) |  |
|  | Unclear (don’t know) | 11 (1.6%) |  |
| **Previous holiday in HRA** | |  |  |
|  | Yes | 139 (45.5%) |  |
|  | No | 161 (52.6%) |  |
|  | Unclear (don’t know) | 6 (2.0%) |  |
| **Planned holiday in HRA** | |  |  |
|  | Yes | 109 (35.6%) |  |
|  | No | 134 (43.8%) |  |
|  | Unclear (don’t know) | 63 (20.6%) |  |
