# Supplementary material for: Using protection motivation theory to explain intentions to vaccinate against tick-borne encephalitis
Source: BMC Public Health. 2025 Dec 23;25:4345. doi: 10.1186/s12889-025-25470-6 (PMC12751593; doi:10.1186/s12889-025-25470-6)
Supplement: Supplementary file 2 — Supplementary Material 2. [file 12889_2025_25470_MOESM2_ESM.docx]

**TBE Questionnaire**

**1. Demographic Data (Betsch et al., 2020)**

Please state your age in whole years.

Please indicate your gender.

• Male

• Female

• Diverse

Are you currently living in Germany?

• Yes

• No

Do you live in the city or in the countryside?

• Rural community (under 5,000 inhabitants)

• Small town (up to 20,000 inhabitants)

• Medium-sized town (up to 100,000 inhabitants)

• Large city (over 100,000 inhabitants)

Please provide information about your educational background.

• Up to 9 years of schooling

• At least 10 years of schooling (without university entrance qualification)

• At least 10 years of schooling (with university entrance qualification)

**2. TBE Information**

You will receive information about the disease TBE (Tick-Borne Encephalitis) and the TBE **vaccination** below. Please read this carefully and calmly. Afterwards, you will be asked to make some assessments.

**3.1 TBE Information KG**

What is TBE?

TBE stands for Tick-Borne Encephalitis and is a flu-like infection. TBE is caused by viruses that are usually transmitted by tick bites. Most cases of TBE occur from spring to autumn.

Symptoms and Course

After a tick bite, flu-like symptoms such as fever, headache, muscle, and joint pain, as well as a general feeling of illness, can occur, usually lasting 2-7 days.

In some cases, a few days after the symptoms have subsided and a temporary improvement, there may be inflammation of the meninges and the brain. Signs of this phase of the illness include recurrent flu-like symptoms and failures of the nervous system. Severe cases can include paralysis of the arms and legs, difficulty swallowing and speaking, respiratory paralysis, and severe drowsiness, which are usually temporary. Deaths from TBE are extremely rare. The majority of infected individuals (approximately 70 to 95%) remain asymptomatic or only experience the initial phase of the illness. Adults and older people usually have more severe symptoms than children.

How can one protect oneself against TBE infection?

Vaccination:

• The Standing Committee on Vaccination (STIKO) recommends the TBE vaccination for all persons who are in risk areas and spend a lot of time in nature.

• Professions such as forestry and agriculture workers.

Avoiding tick bites:

• Ticks are mainly found in undergrowth, bushes, tall grass, and loose foliage.

• Protective clothing and light colors help to detect and avoid ticks more quickly.

• There are also special sprays against ticks that can be applied to the skin. However, their effectiveness is limited in time and does not provide complete protection.

How common is TBE?

In recent years, between 200 and 712 cases of TBE have been reported annually in Germany. In 2020, the highest number of TBE cases since the beginning of data collection in 2001 was recorded, with 712 cases. In 2022, 554 cases of TBE were reported, with the majority of affected individuals either not vaccinated at all or not sufficiently vaccinated.

Where does the TBE virus occur in Germany?

TBE risk areas are defined as districts where cases of TBE occur more frequently than elsewhere. Currently, 178 out of 294 districts in Germany are designated as TBE risk areas.

TBE is mainly found in Bavaria and Baden-Württemberg, but also in southern Hesse, southeastern Thuringia, Saxony, and southeastern Brandenburg. Additional risk areas are sporadically located in central Hesse, Saarland, Rhineland-Palatinate, Lower Saxony, Saxony-Anhalt, and North Rhine-Westphalia.

**4.1 TBE Information GE**

What is TBE?

TBE stands for Tick-Borne Encephalitis and is a flu-like infection. TBE is caused by viruses that are usually transmitted by tick bites. Most cases of TBE occur from spring to autumn.

Symptoms and Course

After a tick bite, flu-like symptoms such as fever, headache, muscle, and joint pain, as well as a general feeling of illness, can occur, usually lasting 2-7 days.

In some cases, a few days after the symptoms have subsided and a temporary improvement, there may be inflammation of the meninges and the brain. Signs of this phase of the illness include recurrent flu-like symptoms and failures of the nervous system. Severe cases can include paralysis of the arms and legs, difficulty swallowing and speaking, respiratory paralysis, and severe drowsiness, which are usually temporary. Deaths from TBE are extremely rare. The majority of infected individuals (approximately 70 to 95%) remain asymptomatic or only experience the initial phase of the illness. Adults and older people usually have more severe symptoms than children.

How can one protect oneself against TBE infection?

Vaccination:

• The Standing Committee on Vaccination (STIKO) recommends the TBE vaccination for all persons who are in risk areas and spend a lot of time in nature.

• Professions such as forestry and agriculture workers.

Avoiding tick bites:

• Ticks are mainly found in undergrowth, bushes, tall grass, and loose foliage.

• Protective clothing and light colors help to detect and avoid ticks more quickly.

• There are also special sprays against ticks that can be applied to the skin. However, their effectiveness is limited in time and does not provide complete protection.

How common is TBE?

In recent years, between 200 and 712 cases of TBE have been reported annually in Germany. In 2020, the highest number of TBE cases since the beginning of data collection in 2001 was recorded, with 712 cases. In 2022, 554 cases of TBE were reported, with the majority of affected individuals either not vaccinated at all or not sufficiently vaccinated.

Where does the TBE virus occur in Germany?

TBE risk areas are defined as districts where cases of TBE occur more frequently than elsewhere. Below, you can see how the number of risk areas in Germany has developed between 2004 and 2023.

FIG: **Bar chart** of TBE risk areas in Germany, as of 2004

FIG: **Bar chart** of TBE risk areas in Germany, as of 2013


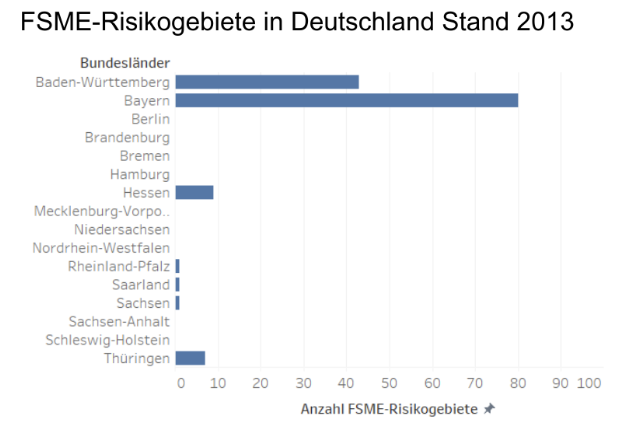


(Example Figure for 2013)

FIG: **Bar chart** of TBE risk areas in Germany, as of 2023

**5.1 TBE Information TA**

What is TBE?

TBE stands for Tick-Borne Encephalitis and is a flu-like infection. TBE is caused by viruses that are usually transmitted by tick bites. Most cases of TBE occur from spring to autumn.

Symptoms and Course

After a tick bite, flu-like symptoms such as fever, headache, muscle, and joint pain, as well as a general feeling of illness, can occur, usually lasting 2-7 days.

In some cases, a few days after the symptoms have subsided and a temporary improvement, there may be inflammation of the meninges and the brain. Signs of this phase of the illness include recurrent flu-like symptoms and failures of the nervous system. Severe cases can include paralysis of the arms and legs, difficulty swallowing and speaking, respiratory paralysis, and severe drowsiness, which are usually temporary. Deaths from TBE are extremely rare. The majority of infected individuals (approximately 70 to 95%) remain asymptomatic or only experience the initial phase of the illness. Adults and older people usually have more severe symptoms than children.

How can one protect oneself against TBE infection?

Vaccination:

• The Standing Committee on Vaccination (STIKO) recommends the TBE vaccination for all persons who are in risk areas and spend a lot of time in nature.

• Professions such as forestry and agriculture workers.

Avoiding tick bites:

• Ticks are mainly found in undergrowth, bushes, tall grass, and loose foliage.

• Protective clothing and light colors help to detect and avoid ticks more quickly.

• There are also special sprays against ticks that can be applied to the skin. However, their effectiveness is limited in time and does not provide complete protection.

How common is TBE?

TBE risk areas are defined as districts where cases of TBE occur more frequently than elsewhere. Below, you can see how the number of risk areas in Germany has developed between 2004 and 2023.

FIG: **Graph** of TBE risk areas in Germany, as of 2004

FIG: **Graph** of TBE risk areas in Germany, as of 2013

FIG: **Graph** of TBE risk areas in Germany, as of 2023


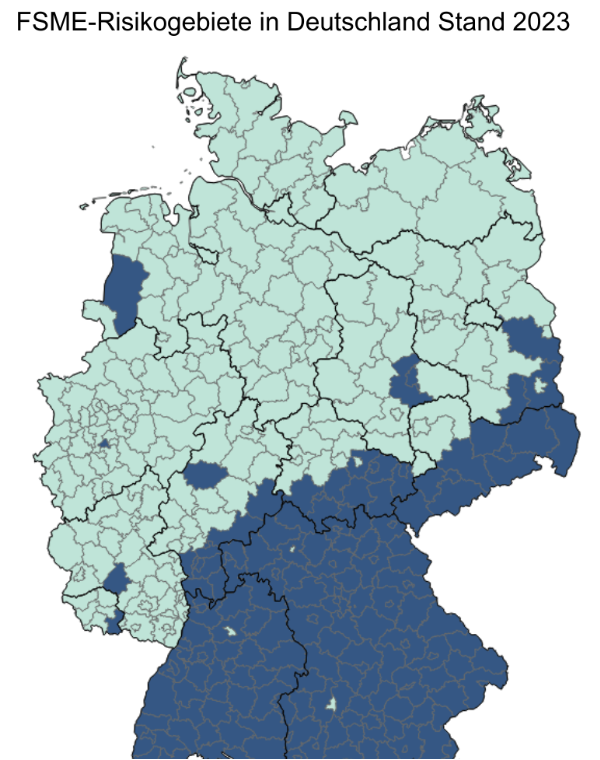


(Example Figure for 2023)

**6. Manipulation Check (Rössler, 2011)**

Thank you for carefully reading the information!

Please rate on a scale how the information was presented. I am interested in your personal opinion.

Uninteresting X X X X X Interesting

Incomprehensible X X X X X Understandable

Not personally relevant X X X X X Personally relevant

Not convincing X X X X X Convincing

Unprofessional X X X X X Professional

Poorly done X X X X X Well done

**7. Protection Motivation Theory (Griffin et al., 2022; Eberhardt & Ling, 2021)**

You will now be asked some follow-up questions.

Please read the following statements and indicate on the scale to what extent you agree or disagree with them. Please note that this is not a test of your knowledge; there are no right or wrong answers. It's about your personal opinion.

Please estimate *(per item):*

Strongly disagree Strongly agree

X X X X X X X

*Items:*

• If I am not vaccinated against TBE, I am at risk of contracting TBE.

• Even if I don't get vaccinated against TBE, I consider it unlikely that I will get TBE.

• If I don't get vaccinated against TBE, I am exposed to the risk of contracting TBE.

• The negative effects of TBE are very serious.

• TBE can be a life-threatening disease.

• TBE is a serious illness for someone like me.

• Not being vaccinated against TBE would have some benefits for me.

• If I don't get vaccinated against TBE, I don't have to worry about the safety of the vaccine.

• If I don't get vaccinated against TBE, I don't have to spend time and effort on vaccination.

• I am confident that an TBE vaccination will effectively reduce my personal risk of contracting TBE.

• An TBE vaccination would protect me from getting TBE.

• An TBE vaccination would guarantee that I do not contract TBE.

• Please select "Strongly agree" to indicate that you have read this question attentively and proceed as usual.

• It would be very easy for me to get vaccinated against TBE.

• It would be difficult for me to get vaccinated against TBE.

• Getting vaccinated against TBE is easy.

• Getting vaccinated against TBE would inconvenience me.

• The TBE vaccination can have side effects (fever, pain, etc.).

• The TBE vaccination can have long-term negative effects on my health.

**8. Vaccination Intention**

Please estimate *(per item)*:

Strongly disagree Strongly agree

X X X X X X X

*items:*

• I am willing to get vaccinated against TBE.

• I plan to get vaccinated against TBE.

• I am likely to get vaccinated against TBE.

**9. Control Variables (Vacc Attitudes: Askelson et al., 2010) (TBE vacc behavior: Erber et al., 2018)**

In the following, you will be asked some questions about your opinions and experiences regarding vaccination and TBE.

Please estimate *(per item)*:

Strongly disagree Strongly agree

X X X X X X X

*items:*

• It is necessary to get vaccinated.

• It is advantageous to get vaccinated.

• It is good to get vaccinated.

Are you already vaccinated against TBE?

• No, I am not vaccinated against TBE.

• Yes, only the first dose.

• Yes, the first and second dose.

• Yes, the first, second, and third doses (primary immunization).

• Yes, the primary immunization and a booster shot.

• Don't know.

Have you had a tick bite in your life?

• Yes

• No

• Don't know

Have you had TBE or another tick-borne disease (e.g., Lyme borreliosis) in your life?

• Yes

• No

• Don't know

**10. Control Variables Leisure (FIFI-K; Nikstat et al., 2018)**

The following questions are about your leisure interests. Please answer the following question.

How often do you engage in the following leisure activities?

You will now be presented with some activities. The "/" are to be understood as "or." Even if you only engage in one of the facts listed under a point, please give your assessment for that activity.

*(per item)*

Never Seldom Sometimes Often Very often

*items:*

• Camping/Tenting

• Climbing/Mountaineering

• Visiting nature (events) and landscapes

• Hiking/Cycling

• Taking walks

• Attending a play/musical/dance performance/opera

• Visiting an art exhibition/museum

• Going on a shopping spree/Shopping

• Watching a series/entertainment show/movie

• Going out with friends/acquaintances

**11. Control Variables Residence (own creation)**

The following is a depiction of the distribution of TBE risk areas in Germany in 2023. Please look at the illustration. Questions related to it will follow.

FIG: Graph of TBE risk areas in Germany, as of 2023

Do you currently live in a risk area?

• Yes

• No

• No response

Have you vacationed in a risk area in the last six months?

• Yes

• No

• Don't know

Do you plan to vacation in a risk area next summer?

• Yes

• No

• Don't know

**12. End Page**

Thank you for participating in the study!

Your answers have been saved. You can now close the browser window.

If you are a student at the University of Erfurt and want to receive 0.25 participant hours, please click on the following link:

For SurveyCircle users: The survey code is: 8831-RF5C-HENX-X8XL

What was the study about?

The aim of the study is to determine which factors influence TBE vaccination intention. Furthermore, I want to find out if the respondents' behavior differs between different groups. For this purpose, some of you received informational material at the beginning in the form of text or with additional presentation of the TBE risk areas in Germany as maps or diagrams. I am interested in whether the way information is presented affects the intention to get vaccinated against TBE.

You can find the information used to create the informational material, as well as further information on the subject of TBE and TBE vaccination, under the following links:

Do you have any questions or comments about this study? Then feel free to contact: [Contact information]

References

Askelson, N. M., Campo, S., Lowe, J. B., Smith, S., Dennis, L. K., & Andsager, J. (2010). Using the Theory of Planned Behavior to Predict Mothers’ Intentions to Vaccinate Their Daughters Against HPV. *The Journal of School Nursing*, *26*(3), 194–202. https://doi.org/10.1177/1059840510366022

Betsch, C., Wieler, L., Bosnjak, M., Ramharter, M., Stollorz, V., Omer, S., ... & Schmid, P. (2020). Germany COVID-19 Snapshot MOnitoring (COSMO Germany): Monitoring knowledge, risk perceptions, preventive behaviours, and public trust in the current coronavirus outbreak in Germany.

Eberhardt, J., & Ling, J. (2021). Predicting COVID-19 vaccination intention using protection motivation theory and conspiracy beliefs. *Vaccine*, *39*(42), 6269–6275. <https://doi.org/10.1016/j.vaccine.2021.09.010>

Erber, W., & Schmitt, H. J. (2018). Self-reported tick-borne encephalitis (TBE) vaccination coverage in Europe: Results from a cross-sectional study. *Ticks and tick-borne diseases*, *9*(4), 768-777.

Griffin, B., Conner, M., & Norman, P. (2022). Applying an extended protection motivation theory to predict Covid-19 vaccination intentions and uptake in 50-64 year olds in the UK. *Social Science & Medicine (1982)*, *298*, 114819. https://doi.org/10.1016/j.socscimed.2022.114819

Nikstat, A., Höft, A., Lehnhardt, J., Hofmann, S., & Kandler, C. (2018). Entwicklung und Validierung einer Kurzversion des Fragebogeninventars für Freizeitinteressen (FIFI-K). *Diagnostica*, *64*(1), 14–25. https://doi.org/10.1026/0012-1924/a000187

Rössler, P. (2011). *Skalenhandbuch Kommunikationswissenschaft*. VS Verlag für Sozialwissenschaften. https://doi.org/10.1007/978-3-531-94179-0
